# The Identification of *Kabatiella zeae* as a Causal Agent of Northern Anthracnose of Sorghum in China and Estimation of Host Resistance

**DOI:** 10.3390/plants13131857

**Published:** 2024-07-05

**Authors:** Wenbo Yu, Yu Wang, Lan Hu, Jing Xu, Jichen Yan, Peng Cao, Chunjuan Liu, Xiaolong Shi, Chang Liu, Yu Jiang, Yufei Zhou

**Affiliations:** 1 College of Agronomy, Shenyang Agriculture University, Shenyang 110866, China; ywb519610@163.com (W.Y.); ws2474456845@gmail.com (Y.W.); liuchunjuan@syau.edu.cn (C.L.); xiaolongshi@syau.edu.cn (X.S.); 2 Liaoning Academy of Agricultural Science, Shenyang 110161, China; laner_lnnky@163.com (L.H.); mljasmine2004@163.com (J.X.); yjc891013@163.com (J.Y.); 3 College of Life and Environmental Science, Wenzhou University, Wenzhou 325035, China; caopeng@wzu.edu.cn

**Keywords:** sorghum northern anthracnose, *Kabatiella zeae*, resistance evaluation, screening of fungicides

## Abstract

Sorghum northern anthracnose is a leaf disease affecting sorghum, which results in plant death and substantial yield loss. This study aimed to effectively understand the disease, clarify its biological characteristics, and evaluate the resistance of germplasm resources. A field sample was collected to isolate and purify the pathogen. The pathogen, identified as *Kabatiella zeae* Narita et Hiratsuka using both morphological and molecular techniques, was further confirmed as the causative agent of northern anthracnose of sorghum following Robert Koch’s principles. The results revealed the optimal culture temperature to be 25 °C, preferred dark culture conditions, and the best growth on potato glucose agar medium with sucrose and L-leucine as the optimal carbon and nitrogen sources, respectively. A total of 138 sorghum germplasm resources were inoculated and evaluated using the isolated pathogen, with 20 lines (14.49%) exhibiting high resistance, 18 lines (13.04%) showing disease resistance, 27 lines (19.57%) demonstrating medium resistance, 37 lines (26.81%) being susceptible, and 36 lines (26.09%) classified as highly susceptible. The indoor fungicide screening was conducted through pathogen medium application, and enilconazole, pyraclostrobin, methylthiophanate, and flusilazole were screened for the best fungicide inhibition with a 100% inhibition rate compared with the control. This study provides reference for field pharmaceutical control in sorghum production.

## 1. Introduction

Sorghum ranks as one of the world’s top five cereals and is a primary choice for agricultural production in arid regions. It plays a significant role in ensuring food and feed security in arid and semi-arid areas globally [1]. In China, sorghum boasts a long-standing history and extensive cultivation, serving as a crucial food and feed crop, as well as a key ingredient in brewing wine and vinegar [2]. However, sorghum faces considerable challenges from biotic and abiotic stresses, with northern anthracnose emerging as a major threat. Northern anthracnose, also known as eye spot disease, is caused by *Kabatiella zeae* Narita et Hiratsuka, and it affects the aerial parts of sorghum, including the stems and leaves. Initially reported in Japan in 1959, the disease primarily infected corn and was identified as a new pathogen, although it did not affect sorghum at that time [3]. However, in 1966, it infected both corn and sorghum in Jilin Province, China [4]. Although the pathogen responsible for northern anthracnose of sorghum is the same as that of northern anthracnose of maize, they are two different host specializations on sorghum and maize, respectively. The recent emergence of northern anthracnose of sorghum in Fuxin City, Liaoning Province, China, poses a significant threat to sorghum production in the country.

Northern anthracnose of maize, caused by *Kabatiella zeae*, has seriously hampered the production of maize globally. Between 2012 and 2015, the grain yield losses due to northern anthracnose in 22 corn-producing areas in the United States and Ontario, Canada, exceeded about 12 million bushels annually [5]. Initially reported in Jilin Province, China, the disease resulted in significant yield losses in maize [4]. The disease subsequently spread to Heilongjiang Province, Liaoning Province, Yunnan Province, and other regions, becoming a primary yield-limiting factor in spring corn-planting areas in China [6]. The natural occurrence of northern anthracnose of maize caused an average yield loss of 9% [7,8]. The disease mostly occurred during July and August, and the low temperatures and rainy conditions exacerbated its severity. Northern anthracnose mainly affects the leaves, leaf sheaths, stems, and grains of both maize and sorghum in northern regions. In the initial stage, small, water-soaked spots appear on the leaves, which gradually enlarge into nearly circular or oval shapes, characterized by a grayish-white center measuring 1–2.5 × 0.5–1.5 mm^2^, with brown or purple edges and a narrow yellow halo. The spot sizes vary depending on plant varieties and environmental conditions, with susceptible plant varieties exhibiting larger spots. Severe cases can lead to extensive necrosis of the mesophyll tissue within days, critically affecting photosynthesis and causing nutrient deficiencies. Premature plant senescence is identified as the root cause of yield decline [9]. Concurrently, the disease reduces the average plant height and exacerbates the occurrence of other diseases such as root rot and stem rot [7].

Recent studies have focused solely on the biological characteristics of the pathogen of maize northern anthracnose, with little investigation into the impact on sorghum. A temperature range between 20 °C and 26 °C is optimal for colony growth and conidia production of the northern anthracnose pathogen in northern corn, with 24 °C being the optimum temperature [10]. Moreover, both oat medium [11] and *Kabatiella zeae* medium are considered as optimal media promoting colony growth and conidia production of the pathogen [7].

Once sorghum leaf disease spreads rapidly, it can decimate all leaves within a few days, resulting in significant losses. The occurrence of northern anthracnose of sorghum is closely linked to climatic conditions, cultivation practices, and variety resistance. Factors such as single planting patterns, inadequate field management, and the absence of disease-resistant varieties contribute to its annual escalation [4]. Therefore, effective control measures for northern anthracnose of sorghum are crucial for improving both quality and yield, necessitating the urgent need for effective screening of pesticides, identifying resistant materials, and popularizing the disease resistance of varieties. At present, studies on northern anthracnose of sorghum are limited. Therefore, in this study, we collected a sample from diseased spots in Fuxin, Liaoning Province, China, identified the pathogen based on morphology and ITS1 sequence information, and investigated the effects of the temperature conditions, light conditions, culture media, and carbon and nitrogen sources on *Kabatiella zeae* mycelium growth. Additionally, the study conducted a variety of resistance identification and pesticide screening procedures to offer insights into field prevention and control strategies against northern anthracnose of sorghum.

## 2. Results

### 2.1. Identification of the Pathogen

#### 2.1.1. Morphology Identification

**Figure 1:**
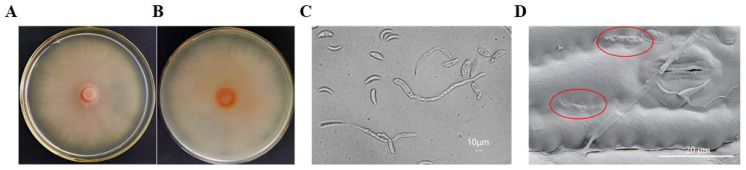
Colony and conidia of *Kabatiella zeae*. (**A**) The adverse vision of mycelial growth of *Kabatiella zeae* on PDA medium after 15 days at temperature of 25 °C; (**B**) the reverse vision of mycelial growth of *Kabatiella zeae* on PDA medium after 15 days at temperature 25 °C; (**C**) conidia morphology under microscope; (**D**) morphology of spores attached to leaves under microscope. The morphology of pathogens on a leaf, as observed under a scanning electron microscope, was highlighted in red.

The colony of the pathogen grew slowly on the PDA medium, initially appearing as milky white. As it aged, the color gradually changed from milky white to pink. The colony displayed a smooth surface, a leathery texture, and radiating ripples, making it challenging to separate it using an inoculation needle on the medium (Figure 1A,B). The hyphae were septate and colorless. Most of the conidia were pointed at both ends, crescent-shaped, and colorless. A few conidia exhibited sickle-shaped, long-spindle-shaped, and short-rod-shaped morphologies. The size range of conidia was 15~31 × 2~4 μm, and the average size was 25.4~3.6 μm. After the diseased leaves were moisturized and cultured, it was observed that conidiophores mostly developed under the host’s stomata. They were very small and light brown and had no bristles (Figure 1C,D).

#### 2.1.2. Molecular Identification

The rDNA-ITS1 sequence of the representative strain BFTJ-1 was sequenced and analyzed, and the results showed that the full length of the ITS1 sequence fragment of the pathogen was 575 bp, and the rDNA-ITS1 sequence of the pathogen was analyzed using GenBank BLAST, which indicated that the sequence of the pathogen was 99.1% homologous to the sequence of *Kabatiella zeae*, which is listed under accession number OQ372991.1 in GenBank.

#### 2.1.3. Pathogenicity

The pathogenicity of the isolated and purified sorghum strain was determined through manual inoculation. Red spots appeared after 7 days of inoculation, followed by the development of round or oval brown lesions after 10 days, exhibiting identical symptoms to those observed in the field. No lesions were found in the control group inoculated with sterile water. The pathogen was subsequently reisolated and identified by morphological examination and rDNA-ITS sequence analysis, confirming its identity in accordance with Koch’s postulates.

### 2.2. Biological Characteristics

#### 2.2.1. Effects of Temperature Conditions on *Kabatiella zeae* Mycelium Growth

**Figure 2:**
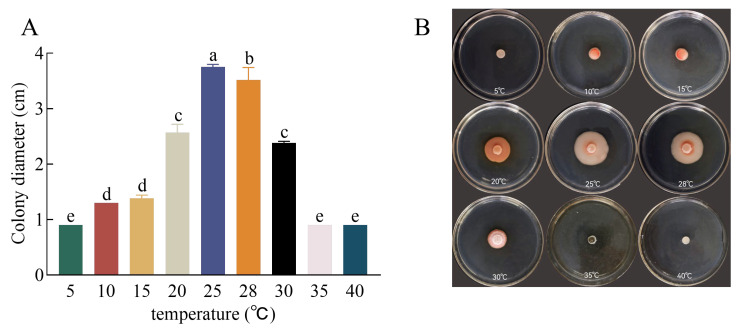
Effects of temperature conditions on the growth of *Kabatiella zeae*. (**A**) Colony diameter of the pathogen grew at different temperatures on PDA medium for 7 days. Different lowercase letters between each bar indicate significant differences among the different temperature (*p* < 0.05); (**B**) morphology of pathogen colonies at different temperatures.

The temperature range for mycelium growth was between 10 °C and 30 °C, with no growth observed at 5 °C, 35 °C, or 40 °C. At 10 °C, the colony diameter reached 1.3 cm (including 0.9 cm of piece diameter), 1.38 cm at 15 °C, and 2.56 cm at 20 °C 7 days after inoculation. The mycelium growth rate increased steadily with an increase in temperature. However, above 25 °C, the growth rate began to decrease gradually. For instance, the colony diameter was 3.61 cm at 28 °C and 2.4 cm at 30 °C, indicating that the optimal temperature for the pathogen’s culture growth was 25 °C (Figure 2).

#### 2.2.2. Effects of Light Conditions on *Kabatiella zeae* Mycelium Growth

**Figure 3:**
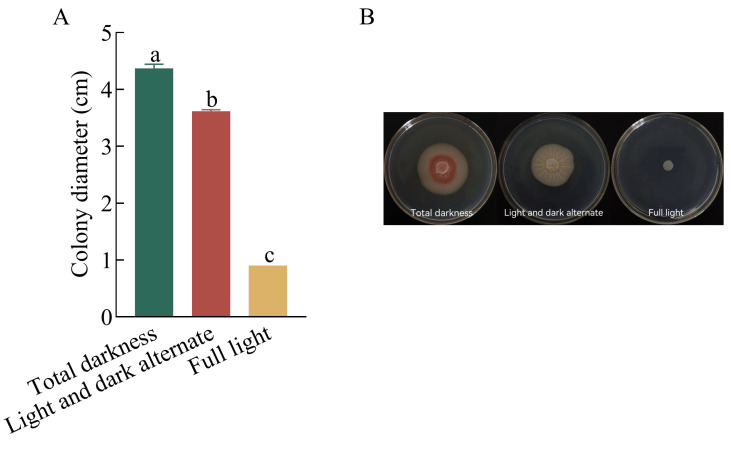
Effects of light conditions on the growth of *Kabatiella zeae*. (**A**) Colony diameter of the pathogen grew under different light conditions on PDA medium at 25 °C for 7 days. Different lowercase letters between each bar indicate significant differences among the different light conditions (*p* < 0.05); (**B**) morphology of pathogen colonies under different light conditions.

Varying illumination conditions were used to detect the most favorable light condition for colony growth. The result presented that the colony diameter reached 4.37 cm in complete darkness. The second highest growth occurred under alternate light and darkness for 12 h, with a colony diameter of 3.62 cm. The pathogen did not exhibit growth under full light conditions, indicating that complete darkness was the most favorable condition for colony growth (Figure 3).

#### 2.2.3. Effects of Culture Medium on *Kabatiella zeae* Mycelium Growth

**Figure 4:**
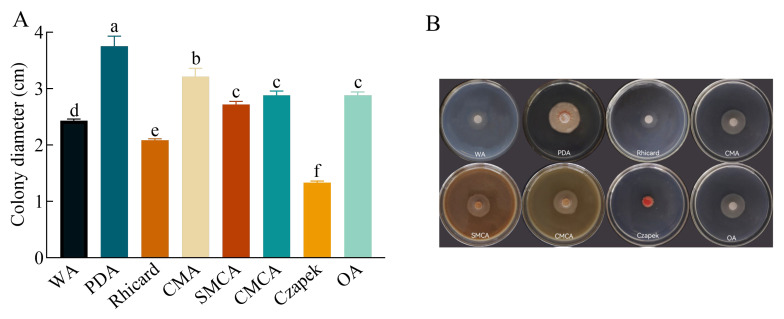
Effect of culture medium on the growth of *Kabatiella zeae*. (**A**) Colony diameters of the pathogen grew on different media at 25 °C for 7 days. Different lowercase letters between each bar indicate significant differences among the different culture media (*p* < 0.05); (**B**) colony morphology of the pathogen in different culture media.

Among the eight media tested, the pathogen exhibited the fastest growth on PDA, with a colony diameter of 3.75 cm. This was followed by CMA with 3.22 cm, OA with 2.88 cm, CMCA with 2.83 cm, SMCA with 2.71 cm, WA with 2.43 cm, and Richard with 2.43 cm. Therefore, it can be concluded that the pathogen thrives best on the PDA medium (Figure 4).

#### 2.2.4. Effects of Carbon and Nitrogen Sources on *Kabatiella zeae* Mycelium Growth

**Figure 5:**
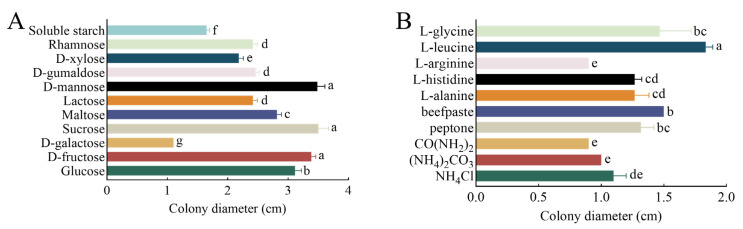
Effects of carbon and nitrogen sources on the growth of *Kabatiella zeae*. (**A**) Diameter of pathogen colony under different carbon source conditions. (**B**) Colony diameter of the pathogen grew under different nitrogen source conditions. All treatments were grown at 25 °C for 7 days. Different lowercase letters between each bar indicate significant differences (*p* < 0.05).

Different carbon sources significantly influence mycelium growth. Among the 11 carbon sources examined, including glucose, D-fructose, D-galactose, sucrose, maltose, lactose, D-mannose, D-gumaldose, D-xylose, rhamnose, and soluble starch, sucrose led to the fastest mycelium growth, with a colony diameter reaching 3.5 cm 7 days after inoculation. D-mannose followed closely, with a colony diameter of 3.48 cm; next, was D-fructose, with a colony diameter of 3.38 cm. Conversely, D-galactose was unsuitable for colony growth, resulting in a diameter of only 1.1 cm (Figure 5A). Among 10 types of nitrogen sources, including NH_4_Cl, (NH_4_)_2_CO_3_, CO(NH_2_)_2_, peptone, beef paste, L-alanine, L-histidine, L-arginine, L-leucine, and L-glycine, the mycelium grew fastest in the medium with L-leucine as the nitrogen source, and the colony diameter reached 1.83 cm. Beef paste ranked the second, with a colony diameter reaching 1.5 cm, followed by L-glycine, with a colony diameter of 1.47 cm. CO(NH_2_)_2_ and L-alanine were not suitable for colony growth, resulting in a diameter of only 0.9 cm (Figure 5B).

### 2.3. Identification of Sorghum Germplasm Resources Resistant to Northern Anthracnose

The results of resistance identification for two consecutive years, from 2022 to 2023, are presented in Table S1. Among the examined 138 sorghum germplasm resources, 20 lines (accounting for 14.49% of total) showed high resistance (HR) to northern anthracnose of sorghum; 18 lines (accounting for 13.04% of total) showed resistance (R); and 27 lines (accounting for 19.57% of total) showed medium resistance (MR). Furthermore, 37 susceptible (S) resources were detected, accounting for 26.81%, and 36 resources were found to be highly susceptible (HS), accounting for 26.09%.

### 2.4. Screening of Indoor Pesticides

**Figure 6:**
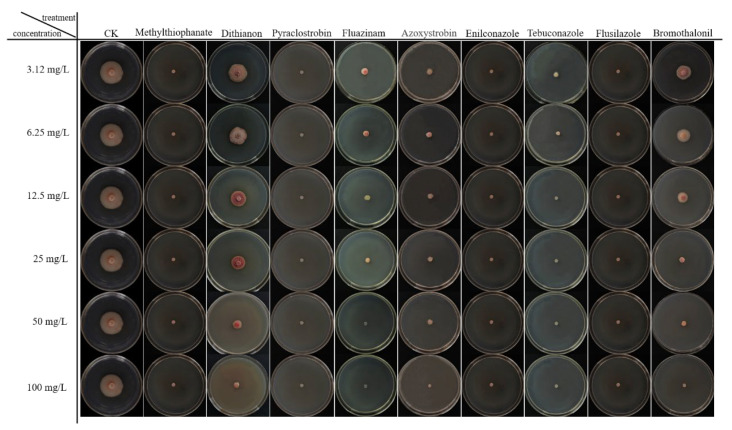
Inhibitory effect of nine different fungicides at six concentrations (from 3.12 to 100 mg/L) on *Kabatiella zeae*. The CK treatment was no fungicide being added to *Kabatiella zeae*. The growth condition was 25 °C for 7 days on PDA medium.

**Figure 7:**
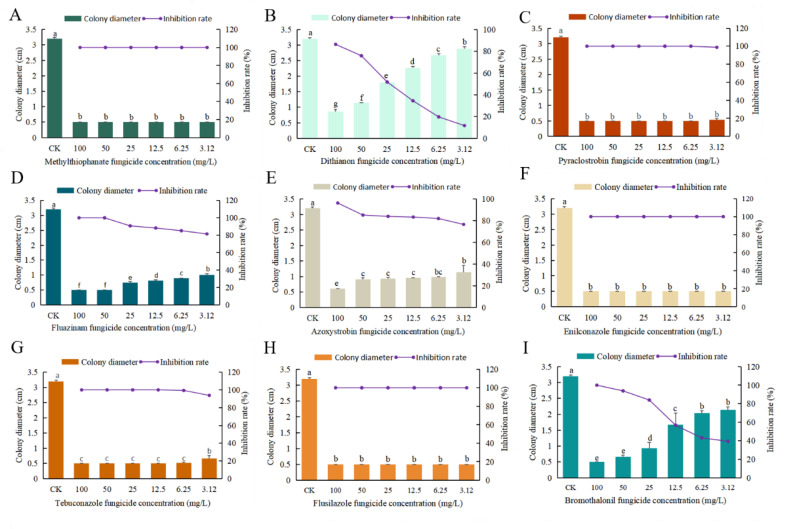
The graphs illustrate the inhibitory effects of various pesticides on northern anthracnose of sorghum. (**A**) Antifungal rate of methylthiophanate. (**B**) Antifungal rate of dithianon. (**C**) Antifungal rate of pyraclostrobin. (**D**) Antifungal rate of fluazinam. (**E**) Antifungal rate of azoxystrobin. (**F**) Antifungal rate of enilconazole. (**G**) Antifungal rate of tebuconazole. (**H**) Antifungal rate of flusilazole. (**I**) Antifungal rate of bromothalonil. The growth condition was 25 °C for 7 days on PDA medium. Different lowercase letters between each bar indicate significant differences (*p* < 0.05).

Enilconazole, pyraclostrobin, methylthiophanate, and flusilazole exhibited the most potent antifungal effects, completely inhibiting the growth of the pathogen at concentrations of 100, 50, 25, 12.5, 6.25, and 3.12 mg/L, with inhibition rates reaching 100% (Figure 6 and Figure 7A,C,F,H). Dithianon showed relatively poor inhibitory effects, achieving a maximum inhibition rate of only 86%, which lacks significant reference value (Figure 7B). Fluazinam demonstrated effective pathogen activity inhibition, with inhibition rates of 100% at concentrations above 50 mg/L, 91% at 25 mg/L, 88% at 12.5 mg/L, 85% at 6.25 mg/L, and 81% at 3.12 mg/L (Figure 7D). Azoxystrobin exhibited inhibition rates of 96%, 86%, 85%, 84%, and 83% at concentrations of 100, 50, 25, 12.5, and 6.25 mg/L, respectively (Figure 7E). Tebuconazole demonstrated effective pathogen activity inhibition, with a colony diameter of 0.6 cm and an inhibition rate of 93% at a concentration of 3.12 mg/L. At other concentrations, no growth was observed, achieving a 100% inhibition rate (Figure 7G). Bromothalonil also exhibited effective pathogen activity inhibition, with inhibition rates of 100%, 94%, 84%, 57%, 43%, and 39% at concentrations of 100, 50, 25, 12.5, 6.25, and 3.12 mg/L, respectively (Figure 7I).

## 3. Discussion

Northern anthracnose is a newly identified disease that is different from Northern anthracnose is a newly identified disease that is different from sorghum diseases, such as target spot disease (*Bipolaris sorghicola*) and leaf blight (*Exserohilum turcicum*). Although it can result in leaf death, its symptoms are markedly distinct. Anthracnose spots, caused by *Colletotrichum sublineola*, appear round or oval with a reddish-brown center and purplish-red, orange, dark purple, or brown edges, often developing small black acervuli later on [12]. Target leaf spot disease manifests as oval, irregular round, oblong or nearly rectangular spots bound by veins, exhibiting distinct concentric bands of light brown and purplish red, resembling an irregular “target ring” [13]. Meanwhile, leaf blight, caused by *Setosphaeria turcica*, typically present as elongated spindle-shaped or oval lesions with a light brown to brown center. In the later stages, small lesions may converge to form a larger lesion, with densely distributed black mold layers on both sides of the lesion [14]. However, the northern anthracnose of sorghum observed in this study appeared as round or oval lesions with a purple-red hue, featuring a grayish-white center and brown or purple edges, often accompanied by a narrow yellowish halo. Dense clusters of these spots can cause the entire leaf to turn red.

Morphological identification relies on the characteristics exhibited by pathogens. Based on the traits observed in the mycelium and the community isolated from sorghum plants, the identification of the fungal pathogen of sorghum anthracnose was preliminarily identified as *Colletotrichum sublineola* by this method [12]. However, this study classified the disease as northern anthracnose of sorghum caused by *Kabatiella zeae*, using a combination of morphological characteristic identification and rDNA-ITS sequence analysis and comparison. Studying the biological characteristics of the pathogen is important for understanding disease occurrence patterns and formulating effective control measures. At present, studies on the biological characteristics of the pathogen of sorghum northern anthracnose are limited. A few studies showed that the optimal growth temperature for the pathogen of northern anthracnose of maize was 25 °C, with PDA medium as the preferred growth medium, α-lactose as the optimal carbon source, and beef powder as the preferred nitrogen source [15]. This study found that *Kabatiella zeae* thrived best at a temperature of 25 °C, with sucrose as the optimal carbon source and L-leucine as the preferred nitrogen source, under dark conditions. The findings of this study aligned with those reported by Meng Lingmin, with slight variations observed in the optimal nitrogen source, possibly due to differences in the pathogen habitats and hosts [15].

In this study, 138 sorghum germplasm resources from both domestic and international sources were inoculated in the field over a span of 2 years. It was observed that varieties exhibiting MR or higher accounted for 47.1% of the tested varieties, whereas the remainder were susceptible varieties. This trend aligned with previous research findings on disease resistance, where susceptible varieties were predominant [16]. During the middle and later stages of sorghum growth, especially under cool and high humidity conditions, the disease proliferated rapidly, leading to premature leaf death and significant yield losses. This posed significant security risks to sorghum production in northeast China. Breeding disease-resistant varieties is considered the most economical and effective measure for disease control, and conducting variety resistance evaluations forms the basis of disease-resistant breeding [17]. Sorghum disease resistance identification experiments have proved that the scientific inoculation method and identification and evaluation systems are crucial for screening germplasms. In the past, sorghum disease resistance identification was mostly based on natural disease intensity in the field [18], but only a few studies have explored disease resistance resources using artificial inoculation technology [19], which has increased the uncertainty of the utilization of resistance resources and led to the slow progress of resistance breeding research. This study made improvements to the culture and inoculation methods for pathogen inoculum, leading to the standardization of sorghum resistance identification technology. This overcame the limitations of using natural diseases to evaluate resource resistance, thereby obtaining more accurate identification results. The fungicides found to have the best control effect in this study were enilconazole, pyraclostrobin, methylthiophanate, and flusilazole. Additionally, azoxystrobinpropiconazole had the best control effect of 18.7%, followed by methylthiophanate with 70% [20]. Methylthiophanate had a good control effect on northern anthracnose of sorghum and maize, but it exhibited a better control effect on sorghum compared to maize, potentially due to differences in the hosts.

This study investigated the biological characteristics of the pathogen of sorghum northern anthracnose, elucidating the pathogen’s nutritional and environmental requirements and adaptability. These findings provided a basis for further studies on the occurrence patterns of diseases. In addition, the disease resistance of 138 sorghum germplasm resources was assessed over a span of 2 years. The results revealed that 20 sorghum germplasm resources exhibited HR and 36 exhibited HS to the disease. This expanded the pool of disease-resistant resources for northern anthracnose of sorghum through indoor screening of four fungicides, enilconazole, pyraclostrobin, methylthiophanate, and flusilazole, which were identified as having superior pathogen inhibition effects, laying the groundwork for chemical control of northern anthracnose of sorghum.

## 4. Materials and Methods

### 4.1. Sample Collection, Isolation, and Preservation of the Pathogen

The sample of one infected plant was collected from Fuxin, Liaoning Province, China, and the pathogen was isolated and purified using the tissue separation method [21]. Small pieces measuring 5 mm^2^ were excised from the junction of diseased leaves of the standard samples, sterilized with 70% ethanol for 3 min, and then cultured in the dark on PDA plates (9 cm in diameter) at 25 °C to obtain the isolate. The PDA medium was provided by LvYuan Biochemistry Company. It consists of peeled potatoes, glucose, and agarose. The purified strain was obtained according to single spore isolation. After 7 days of culture on the PDA plates, the colony morphology and conidia shape and size were observed and recorded. The representative strain was named BFTJ-1 and preserved at the Institute of Plant Protection, Liaoning Academy of Agricultural Sciences.

### 4.2. Identification of the Pathogen

#### 4.2.1. Morphological Observation

The isolated pathogen was inoculated onto sterile PDA plates and cultured in the dark at 28 °C for 7 days. The colony morphology, color, and texture were observed and recorded. The morphological characteristics of the conidia were examined under a microscope using slide culture, which served as the main morphological basis for identifying the pathogen.

#### 4.2.2. Molecular Biological Identification

The rDNA of the fungus was extracted by the CTAB method [22] and amplified by PCR using universal primers for the transcribed spacer (ITS1) region of the fungal ribosomal gene [23]. The PCR reaction mixture consisted of 20 μL, including 10 μL of 2 × Taq PCR Mix (TianGen Biotech Co., Ltd., Beijing, China), 1 μL of total rDNA template, 0.8 μL of 10 μmol·L^−1^ upstream and downstream primers each, and 7.6 μL of ddH_2_O. The PCR amplification reaction program involved preheating at 94 °C for 4 min, followed by denaturation at 94 °C for 1 min, annealing at 55 °C/62 °C for 30 s, extension at 72 °C for 30 s for 35 cycles, final extension at 72 °C for 10 min, and then storage at 4 °C. The obtained PCR products were sequenced by Dingguo Biotechnology Co., Ltd. and compared with the existing information in GenBank.

#### 4.2.3. Pathogenicity Determination

The obtained single colony strains were inoculated into the potato dextrose (PD) liquid culture medium and cultured in a shaking table at 140 rpm and 28 °C for 72 h in the dark. A spore suspension with a concentration of 1 × 10^5^ spores/mL was prepared using sterile water and inoculated onto 10 sorghum seedlings using an artificial inoculation method [16]. Simultaneously, normal cultivation practices were conducted as a control, with inoculation using clear water. Once the plants exhibited symptoms of disease, the pathogen was isolated and purified again [21]. Morphological and molecular biological identifications were performed to confirm whether it matched the inoculum, thereby completing Robert Koch’s postulate verification process.

### 4.3. Study on Biological Characteristics

#### 4.3.1. Effects of Temperature Conditions on *Kabatiella zeae* Mycelium Growth

*Kabatiella zeae* pieces, 9 mm in diameter, were inoculated onto the PDA culture medium using a punch. They were then incubated in the dark at various temperatures: 5 °C, 10 °C, 15 °C, 20 °C, 25 °C, 28 °C, 30 °C, 35 °C, and 40 °C. Each treatment was replicated three times, and the colony diameter was measured using the cross method after 7 days.

#### 4.3.2. Effects of Light Conditions on *Kabatiella zeae* Mycelium Growth

A 9 mm piece of *Kabatiella zeae* was inoculated onto the PDA culture medium. Three treatments were established in an artificial climate box: continuous darkness for 24 h, alternating light and darkness for 12 h, and continuous illumination for 24 h. Each treatment was cultured at 25 °C, and the colony diameter was measured using the cross method after 7 days.

#### 4.3.3. Effects of Culture Medium on *Kabatiella zeae* Mycelium Growth

The pathogen was inoculated into water agar medium (WA), potato dextrose agar medium (PDA), Richard medium (Richard), cornflour medium (CMA), sorghum leaf medium (SMCA), corn leaf medium (CMCA), Czapek–Dox medium (Czapek) and oatmeal agar medium (OMA), respectively, by using a hole punch with a diameter of 9 mm and then cultured in the dark at 25 °C for 7 days [24].

#### 4.3.4. Effects of Carbon and Nitrogen Sources on Mycelium Growth

Richard was used as the base culture medium, and the carbon source was substituted with glucose, D-fructose, D-galactose, sucrose, maltose, lactose, D-mannose, D-gumaldos, D-xylose, rhamnose, and soluble starch. The nitrogen sources were replaced with NH_4_Cl, (NH_4_)_2_CO_3_, CO(NH_2_)_2_, peptone, beef paste, L-alanine, L-histidine, L-arginine, L-leucine, and L-glycine in equal amounts. Each treatment was repeated three times and cultured in the dark at 25 °C. After 7 days, the cross method was used for measurement.

### 4.4. Evaluation of Disease Resistance of Germplasm Resources

#### 4.4.1. Evaluation of Sorghum Germplasm Resources

A total of 138 sorghum germplasm resources were examined, including 101 from China, 12 from the United States, 22 from India, and 3 from Mexico.

#### 4.4.2. Preparation of Inoculum

The BFTJ-1 strain (evaluated strains of sorghum northern anthracnose) were transferred onto the PDA culture medium and incubated at 25 °C for 3–5 days. Then, the pathogen blocks were transferred onto the PD culture medium and cultured at 25 °C for 3 days. A spore suspension was prepared with a concentration of 1 × 10^5^/mL using sterile water, and 0.1% Tween 20 was added for later use.

#### 4.4.3. Inoculation Method

**Figure 8:**
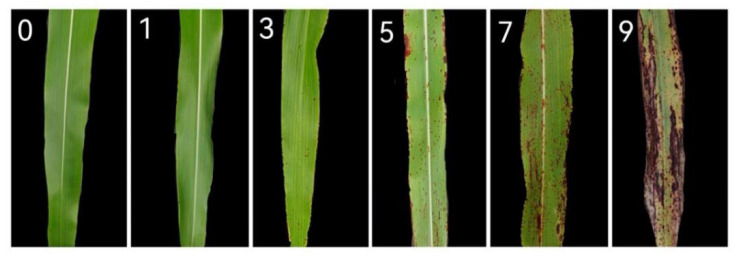
Symptoms of different disease grades on diseased leaves. The number in this figure presented the rating scale of the diseased leaves.

The experimental site is situated in the experimental field of Liaoyang Institute of Economic Crops, Liaoning Academy of Agricultural Sciences (41.37 N, 123.86 E). Sorghum disease resistance was assessed for two consecutive years, from 2022 to 2023, through artificial inoculation in the field, with planting typically occurring in late April. Each plot consisted of two rows, each 5 m in length, with a row spacing of 0.6 m. One seedling was left in each hole, totaling about 40 seedlings per area. The experiment was repeated twice during the 2 years. The inoculation took place at the trumpet stage of the plant, The inoculation took place at the trumpet stage of the plant. Specifically, It was 10-leaf to 11-leaf stage for middle- and late-maturing varieties, and the 9-leaf stage for early-maturing varieties. Evening or rainy days were preferred for inoculation. The spray inoculation method was employed, with spore suspension evenly sprayed onto the leaf surfaces around the trumpet mouth of the plant. The amount of suspension applied was adjusted to ensure even coverage without excess droplets. Water spray and moisture retention were facilitated within 48 h post-inoculation to increase pathogen infection. Approximately 40–50 days after inoculation, the sorghum seeds entered the maturity stage, and disease investigation commenced. Emphasis was placed on investigating the upper and lower leaves at the inoculation site. Each leaf was inspected individually according to the disease identification standards (Figure 8), as outlined in Table 1. The incidence level was calculated, and the resistance level of the identified materials was determined based on the disease severity. The evaluation of disease resistance was conducted based on the results obtained over the 2-year period.

### 4.5. Indoor Screening of Fungicides

#### 4.5.1. Test Reagent

The following test reagents were used in this study: 250 g/L of pyraclostrobin (Zhejiang Hangzhou Yulong Chemical Co., Ltd., Hangzhou, China); 250 g/L of azoxystrobin (Syngenta Nantong Crop Protection Co., Ltd., Nantong, China); 430 g/L of tebuconazole (Bayer Crop Science Co., Ltd., Leverkusen, Germany); 22.7% dicyanoanthraquinone (Jiangxi Heyi Chemical Co., Ltd., Jiujiang, China); 500 g/L of fluazinam (Ishihara Industrial Co., Ltd., Higashiosaka, Japan); 20% imazalil (Jiangxi Heyi Chemical Co., Ltd., Jiujiang, China); 40% fluorosilicone (Jiangsu Jiannong Plant Protection Co., Ltd., Weifang, China); 500 g/L of thiophanate methyl (Chengdu Kelilong Biochemical Co., Ltd., Chengdu, China); and 25% bromothalonil (Jiangsu Tuoqiu Agrochemical Co., Ltd., Yancheng, China).

#### 4.5.2. Screening Method

Nine fungicides were evaluated under laboratory conditions using the mycelium growth rate method [25]. The fungicides were diluted to concentrations of 100, 50, 25, 12.5, 6.25, and 3.125 mg/L, respectively. These diluted fungicides were then added to the PDA culture medium, which was melted after sterilization beforehand, resulting in a medicated culture medium with a drug-to-medium ratio of 1:9. The mixture was poured into Petri dishes and allowed to cool. After cooling, a number of pathogenic pieces were punched into the prepared pathogenic culture dish using a 5 mm punch, and one pathogenic piece was placed at the center of each medicated culture dish. After being cultured in a constant temperature incubator at 25 °C for 7 days, the diameter of the colonies was measured using the cross method, and the inhibition rate of the drugs against the pathogen was calculated.

$$\mathrm{Inhibition}\;\mathrm{rate}=\frac{\mathrm{Control}\;\mathrm{colony}\;\mathrm{diameter}-\mathrm{Treated}\;\mathrm{colony}\;\mathrm{diameter}}{\mathrm{Control}\;\mathrm{colony}\;\mathrm{diameter}-\mathrm{Piece}\;\mathrm{diameter}}\times 100\%$$

### 4.6. Statistical Analysis

All the data were analyzed using one-way ANOVA and multiple comparison between different treatments was calculated using Duncan’s test in the software IBM SPSS 26.0. Different letters indicate a significant difference at *p* ≤ 0.05. The data were plotted using GraphPad Prims 8 software.

## Figures and Tables

**Table 1:** Identification and evaluation criteria for sorghum germplasm resources resistant to northern anthracnose of sorghum. HR, high resistance; R, resistant; MR, medium resistance; S, susceptible; HS, highly susceptible.

Rating Scale	Resistance Evaluation	Description of Disease Symptom
1	HR	Only a few chlorotic flecks were present on the inoculated leaves, but no acervuli were visible. Lesions covered less than 5% of the leaf surface.
3	R	Some circular to oval spots and acervuli development were observed. Lesions covered 5.1–20% of the leaf surface.
5	MR	Abundant circular to oval spots on inoculated leaves and black acervuli development were observed. Lesions covered 20.1–40% of the leaf surface.
7	S	The leaf area was found to be covered with coalescing lesions with black acervuli. Lesions covered 40.1–70% of the leaf surface.
9	HS	Lesions joined to cover a large proportion of the leaf surface with abundant black acervuli. The whole plants were almost dead. Lesions covered more than 70.1% of the leaf surface.

## Data Availability

The data that support the findings of this study are available from the corresponding author upon reasonable request.

## Supplementary Materials

The following supporting information can be downloaded at https://www.mdpi.com/article/10.3390/plants13131857/s1. Table S1: Identification results of northern anthracnose of sorghum inoculation.
