# Supplementary material for: The Identification of Kabatiella zeae as a Causal Agent of Northern Anthracnose of Sorghum in China and Estimation of Host Resistance
Source: Plants (Basel). 2024 Jul 5;13(13):1857. doi: 10.3390/plants13131857 (PMC11244406; doi:10.3390/plants13131857)
Supplement: Supplementary file 1 [file plants-13-01857-s001.zip › plants-3058183-supplementary.pdf]

Supplementary Materials

Table S1. Identification results of northern anthracnose of sorghum inoculation.

No.	Variety	Source	Progression	Rating	No.	Variety	Source	Progression	Rating
1	bendigaoliangzhe	China	9	HS	70	dongshanhonggaoliang	China	3	R
2	daluochuigaoliang	China	7	S	71	bayeqi	China	7	S
3	dahongsheyan	China	7	S	72	hongkebainiangaoliang	China	7	S
4	erniuxin	China	5	MR	73	xiaolaojiaozi	China	5	MR
5	baodizu	China	7	S	74	erniuxin	China	7	S
6	gaoliang	China	5	MR	75	baigaoliang	China	7	S
7	dabaigaoliang	China	5	MR	76	yidumigaoliang	China	5	MR
8	dasheyan	China	7	S	77	baluocha	China	5	MR
9	xaionianbang	China	9	HS	78	hongke	China	5	MR
10	mugewo	China	9	HS	79	hongqida	China	7	S
11	sanhuangke	China	9	HS	80	kuchehuang	China	7	S
12	yankou	China	9	HS	81	ermaokui	China	7	S
13	tiangaoliang	China	7	S	82	dalihong	China	9	HS
14	zhuyeqing	China	5	MR	83	gaohushu	China	3	R
15	qisiwugaoliang	China	5	MR	84	aijiaohuang	China	5	MR
16	sansuigaoliang	China	9	HS	85	changsui	China	5	MR
17	tiangaoliang	China	7	S	86	heilaopofanbaiyan	China	9	HS
18	changtinggangoaliang	China	5	MR	87	qisiwu	China	5	MR
19	dachuiluo	China	9	HS	88	sanbianse	China	7	S
20	hongjiaozi	China	5	MR	89	tianganshushu	China	7	S
21	dachuiluo	China	7	S	90	huangkefangaoliang	China	7	S
22	jiuyezi	China	9	HS	91	erehuangjin	China	9	HS
23	cuobazi	China	9	HS	92	tiangaoliang	China	9	HS
24	huangjingaoliang	China	9	HS	93	ganzhezi	China	9	HS
25	qingebaigaoliang	China	7	S	94	guandongqing	China	7	S
26	xiaogouyaruozi	China	9	HS	95	tiangaoliang	China	5	MR
27	ganshuacao	China	9	HS	96	tiangaoliang	China	5	MR
28	yankoushushu	China	9	HS	97	heigaoliang	China	7	S
29	qingkelimaji	China	9	HS	98	fangaoliang	China	3	R
30	xiaohongmiliang	China	5	MR	99	liantangai	China	7	S
31	jiuyeqi	China	7	S	100	tiangaoliang	China	7	S
32	hongkebangzi	China	9	HS	101	dabaigaoliang	China	9	HS
33	daehuang	China	9	HS	102	TxR2356	America	3	R
34	xiaohuangjiao	China	7	S	103	America	America	5	MR
35	migaoliang	China	7	S	104	MN-4407	America	1	HR
36	yanzhihong	China	7	S	105	BEAYER	America	3	R
37	huangluosan	China	5	MR	106	IS-1139C	America	5	MR
38	tianluli	China	7	S	107	TX622B	America	3	R
39	youxinhu	China	9	HS	108	P-932149	America	1	HR
40	dahongpao	China	7	S	109	Drougt 7	America	9	HS

Table S1. (continued)

No.	Variety	Source	Progression	Rating	No.	Variety	Source	Progression	Rating
41	dabeihonggoaliang	China	9	HS	110	TxR8508	America	1	HR
42	heikesheyan	China	9	HS	111	KAPIRE	America	9	HS
43	daqingke	China	9	HS	112	MN-839	America	1	HR
44	guandongqing	China	9	HS	113	QUERY3	America	3	R
45	hongliuzi	China	9	HS	114	ICSV-153	India	1	HR
46	hongwobai	China	9	HS	115	SPV472	India	7	S
47	dahongpao	China	7	S	116	E92	India	5	MR
48	dahongpao	China	7	S	117	168	India	3	R
49	qisiwu	China	7	HS	118	IS-3784	India	5	MR
50	lvyuexian	China	7	HS	119	IS-3977	India	3	R
51	tianluli	China	7	HS	120	A16009	India	3	R
52	dawaitou	China	3	R	121	India	India	9	HS
53	huangniangaoliang	China	5	MR	122	IS-2327	India	1	HR
54	honggaoliang	China	7	S	123	0-1	India	3	R
55	jindinghong	China	7	S	124	A717	India	5	MR
56	dahongpao	China	3	R	125	ICSH106	India	1	HR
57	qiongyue253	China	5	MR	126	PS21663	India	1	HR
58	huangkeben	China	5	MR	127	ICSV739	India	1	HR
59	huangniangaoliang	China	7	S	128	ICSB15	India	1	HR
60	houziyan	China	9	HS	129	ICSV364	India	1	HR
61	dahongpao	China	7	S	130	ICSV685	India	1	HR
62	damaoruo	China	7	S	131	MR-878	India	1	HR
63	badaye	China	5	MR	132	A701(80R)	India	1	HR
64	tiangaoliang	China	3	R	133	A504(80R)	India	1	HR
65	qiongyue360	China	7	S	134	M55812	India	1	HR
66	honggaoliangjie	China	1	HR	135	M-67064	India	1	HR
67	bainiangaoliang	China	0	HR	136	ZACP-1396MB	Mexico	3	R
68	baiping	China	3	R	137	M-40079B	Mexico	1	HR
69	fenzhidahongsui	China	3	R	138	Tx2761(red seed)	Mexico	3	R
